# Impact of Neurolens Use on the Quality of Life in Individuals With Headaches: A Randomized Double-Masked, Cross-Over Clinical Trial

**DOI:** 10.1167/tvst.13.1.27

**Published:** 2024-01-30

**Authors:** Vivek Labhishetty, Jesus Cortes, Corina van de Pol, Ville Maanpaa, Aric Plumley, Neha Amin, Jason Hurley, Tausha Barton, Troy White, Rob Szeliga, J Mathis Dixon, David Grosswald, Jon Knutson, Heavin Maier

**Affiliations:** 1 Neurolens Inc., Costa Mesa, CA, USA; 2 Southern California College of Optometry, Fullerton, CA, USA; 3 Advanced Vision & Achievement Center, Phoenix, AZ, USA; 4 Eyecare of Rigby, Rigby, ID, USA; 5 Buckeye Family Eye Clinic, Hillsboro, OH, USA; 6 Kapperman, White and McGarvey Eyecare, Chattanooga, TN, USA; 7 Spring Hill Eyecare, PLLC, Spring Hill, TN, USA; 8 Advanced Eyecare Center, Perry, GA, USA; 9 The EyeCenter, Conyers, GA, USA; 10 Signature Eye Care, Lincoln, NE, USA; 11 Eyes for Life, Spokane, WA, USA

**Keywords:** digital eyestrain, headache, Neurolens, binocular vision, vergence

## Abstract

**Purpose:**

Vision-related disorders, such as refractive errors and binocular vision issues, can cause headaches. The current study evaluates the impact of Neurolens (NL) on individuals with headaches, assessed using the Headache Impact Test (HIT) questionnaire.

**Methods:**

Subjects (18–60 years) with good stereoacuity and a HIT score of ≥56 points were enrolled. Each subject wore both control lens and NL for 30 ± 10 days each. The primary outcome of the study was to assess the difference in the HIT score between the two treatments.

**Results:**

Of the subjects randomized, 88% (170/195) completed the study. Overall, subjects reported a greater improvement in HIT score improvement with NL compared with control (mean difference, −1.53 points; 95% confidence interval, −2.8 to −0.26; *P* = 0.01). In the subgroup with reduced NPC, subjects reported a larger improvement in HIT score improvement with NL but was not statistically significant (mean difference, −1.89 points; 95% confidence interval, −4.27 to −0.47; *P* = 0.11).

**Conclusions:**

NL produced a statistically significant decrease in the impact of headaches on individuals’ quality of life compared with placebo. Although the overall magnitude of the decrease was not clinically significant, a clinically meaningful improvement with NL cannot be ruled out with high certainty in the current study.

**Translational Relevance:**

Headache is one of the most experienced symptoms by individuals worldwide with vision-related disorders being a primary reason. It is, therefore, critical to screen these disorders before providing a pharmacological intervention, which may have side effects. NL provides an objective way to diagnose and treat digital eyestrain-related headaches.

## Introduction

Headache is one of the most commonly experienced symptoms reported by individuals worldwide. A recent review on the global prevalence of headaches reported an estimated prevalence of approximately 52%*.*^1^ It is also one of the most reported symptoms by patients at primary care offices.^2^ These headaches have been reported to have a debilitating impact on productivity and quality of life of the individual.^3^^,^^4^ There are several reasons why individuals could experience headaches, one of the primary causes being vision related. Uncorrected refractive errors and binocular vision conditions are the major causes of vision-related headaches.^5^ Individuals spend approximately 8 to 12 hours a day on average on digital technology, including phones, tablets, and laptops or desktops.^6^^,^^7^ Sustained near viewing creates an increased demand on the eyes, which could lead to visual fatigue, which then manifests as headaches, eye strain, or tired eyes. A previous study reported that the magnitude of visual symptoms is significantly higher when viewing digital displays when compared with hard-copy printed materials.^8^ This association between vision-related issues and digital usage is commonly referred to as computer vision syndrome (CVS), digital eyestrain, or sometimes even digital vision syndrome.^6^^,^^7^^,^^9^ Digital usage has increased significantly worldwide with approximately 80% of adults reporting some sort of a CVS-related symptom. Individuals with headache-related disorders typically resort to pharmacological interventions, which may or may not comprehensively treat the symptoms and may result in intolerable side effects.^10^ Typically, headache is a self-reported symptom and, depending on the pattern of the symptoms reported (medical history/ questionnaires), clinicians use appropriate diagnostic modalities and/or interventions. However, headaches secondary to vision-related causes are often overlooked and, in many cases, could be addressed using simple nonpharmacological solutions. Therefore, it is important to screen for these vision-related issues in a patient with headaches.

Uncorrected refractive errors can be corrected easily using corrective spectacles or contact lenses, which provide the best possible visual acuity. Binocular vision problems are more difficult to address given the complexity in accurately identifying and clinically diagnosing individuals who could benefit from an intervention. Several treatment options including refractive lenses, prisms,^11^^,^^12^ and vision therapy^13^^,^^14^ are available and often prescribed based on the information obtained from the clinical testing. Although there is no agreement on the best treatment approach for patients with a binocular vision problem, most studies suggest vision therapy (home + office based) as the most effective treatment.^15^ It is critical to note that there several limitations that may impact the prognosis with vision therapy, including the patient's motivation and compliance, economic burden, and the time involved.^16^^,^^17^ Studies that have evaluated the efficacy of prism therapy reported contradictory results and were inconclusive.^12^^,^^22^

To evaluate how well a patient's eyes work together, measurements of accommodation and vergence such as the near point of accommodation, near point of convergence (NPC), negative and positive fusional vergence, or eye misalignment measurements are typically performed. Tests such as prism cover test, Von Graefe or modified Thorington are commonly used to measure eye alignment (vergence) accuracies, clinically termed as phorias. If an individual with significant phoria is symptomatic, treatment options aimed at decreasing the phoria and/or relieving symptoms are recommended.^18^ The current testing routine for phoria estimations, however, is not ideal and has several sources that could cause errors in diagnosing the problem. These sources include the subjective nature of testing (responsiveness of the patient or expertise of the doctor), poor interexaminer repeatability,^19^^–^^21^ and the variability and complexity involved in prescription guidelines like Sheard's or Percival's criterion.^22^ A recent study also reported that the clinical measures, such as Sheard's criterion or fusional vergence magnitude do not tend to correlate with the severity of the symptoms experienced.^23^ This would mean that there is a good chance that the existing clinical routine might miss symptomatic individuals with digital eyestrain who do not fit the diagnostic criterion, but could benefit from an intervention.

### Neurolens (NL) Technology

The NL system consists of a novel device and lens technology that can objectively detect and correct eye misalignment with contoured prism lenses. NL process involves three major steps of detection, evaluation, and treatment of binocular vision issues. The process includes a symptom screener, known as the lifestyle index, a NL Measurement Device, and a novel contoured prism technology that can be applied to the patient's prescriptive lenses. The lifestyle index is a Likert scale-type questionnaire. It contains seven symptom questions (headaches, stiffness/pain in the neck, discomfort with digital use, tired eyes, dry eye sensation, light sensitivity, and dizziness) and the subject rates the frequency of each of the seven symptoms. Response options include never, rarely, sometimes, very often, and always, which are numerical graded as 1 to 5. NL commercially recommends a measurement and intervention if there is a 3+ score on any of the first three symptoms (headaches, stiffness/pain in the neck, or discomfort with digital use) in the questionnaire.

The NL measurement device is a commercially available medical device. The NL measurement device uses an objective technique to assess eye misalignment at distance (6 m) and near (50 cm). It combines a stereoscopic display with cameras which continuously monitor the patient's Purkinje reflection (P1) and the pupil. There are three measurement aspects involved, pupillary distance measurement; dissociated phoria test; and an associated phoria test (Table A1). Based on the measurements, NL measurement device uses a proprietary iterative algorithm to calculate a prescribing guideline, called the Neurolens value.

The Neurolens value is then used to prescribe Neurolenses, which incorporate a proprietary progressive contoured prism into the prescriptive spectacle correction lens design. The Neurolens value calculated in the device represents the prism correction provided at distance. The prism is progressed to provide an additional 0.75 base-in prism at near. This design allows clinicians to provide a variable prism power for different viewing distances.

### Purpose

Vision-related issues cause headaches in this modern-day digital world.^24^^,^^25^ Unlike uncorrected refractive errors, binocular vision problems such as eye misalignments are difficult to correct. The current diagnostic tools are limited in their ability to accurately identify and diagnose binocular vision-related issues. As far as we know, there are also no clinically proven treatment options that reduce the impact of headaches secondary to binocular vision issues. The current study evaluates the efficacy of NL in improving the quality of life of subjects with symptomatic headaches.

## Materials and Methods

This study followed the tenets of Declaration of Helsinki. All study subjects were provided with an informed consent and enrolled only if they were willing to participate and sign the consent form. The study was approved by the Western-Copernicus Group Institutional Review Board, an independent review board (WCG IRB–20215028). This study is also a registered clinical trial (clinicaltrials.gov: NCT05070767). Headache Impact Test (HIT-6) questionnaire was used to assess subject outcomes in the study. We used a double masked cross-over design. The subjects were randomized to either wearing control (Shamir Autograph II single vision or Progressive-addition Lenses [PALs] with Crizal rock) or treatment (NL single vision or PAL with premium antireflection coating) lenses and then crossed over to the opposite lens. Subjects wore each lens for 30 ± 10 days and completed the HIT-6 questionnaire at the end of each visit. Depending on the age and their need for a near add, subjects either got a single vision lens or a PAL. Subjects were recruited from 10 optometry locations across the United States between October and December 2021, with all visits completed by May 2022. Each subject was informed that they were tested using two different spectacle designs to treat their headaches and neither the subject nor the investigator was aware of the treatment order (Table 1).

**Table 1.** Inclusion and Exclusion Criteria


**Inclusion Criteria**
• Age range: 18–60 years of age at the time of signing the informed consent.
• Best-corrected distance and near acuity must be equal to or better than 20/25 Snellen Equivalent in each eye.
• Symptomatic as indicated by the HIT-6 questionnaire (score of ≥56)
• Updated distance spectacle prescription must match the following,
a. Spherical power inclusive between +4.00D to −8.00 D
b. Cylinder power no more than −4.00D cyl
c. ADD power i. Subgroup 1: No ADD ii. Subgroup 2: minimum +1.00D ADD.
• Subjects’ eye alignment tests must match the following:
a. Successful measurement on the NL Measurement Device (Acceptable measurement quality index and a numerical NL value, no low measurement quality index or convergence excess)
• Minimum stereo vision of 50 seconds of arc at 16 inches
**Exclusion Criteria**
• Subjects who need a vertical prism.
• Previously has worn NLs.
• Subjects who need a near of <1.00 D
• Use of contact lenses during the study
• Lack of binocular vision, including strabismus, amblyopia, or suppression
• >20 prism diopter of eye misalignment
• Aniseikonia of >3.00 D spherical equivalent difference between eyes
• Prior ocular surgery that in the estimation of the practitioner induces corneal scarring (radial keratotomy, corneal transplant, etc.) or prior surgery involving the extraocular muscles (strabismus surgery); surgeries that do not affect these parameters such as LASIK, PRK, or pterygium surgery are allowed
• Anterior segment conditions that could obfuscate or obscure reflections from the cornea, or reduce visual acuity, including but not limited to corneal scarring, large pinguecula, pterygium, keratoconus, or cataract
• Clinical dry eye (defined as tear break-up time of <5 seconds)
• Intraocular pressures of >25 mm Hg in either eye or uncontrolled glaucoma
• Macular disease, or any posterior segment finding which in the opinion of the investigator is visually and/or clinically significant
• Change in acute or prophylactic migraine treatment medication or dosage within the previous 2 months
• Previous head or neck trauma (for instance, car accident, etc) requiring medical intervention
• Physical tremors or history of seizures or seizure disorder
• Women who are pregnant or lactating at the time of the study entry
• Mental incapacity that prevents a subject from being able to follow simple instructions such as, “Look at the target”

### Data Collection and Outcome Measures

#### HIT-6 Questionnaire

The HIT-6 is a validated Likert-type questionnaire, typically used to assess the impact of headaches on the quality of life of a symptomatic individual (Table A4).^26^ It contains six questions that capture the impact of headaches. Responses and their corresponding relative weights were as follows: never (6 points), rarely (8 points), sometimes (10 points), very often (11 points), and always (13 points). The HIT-6 survey score is obtained by simply adding the scores of the six questions. The final HIT score can range between 36 and 78. The higher the score, the greater the impact of symptoms on an individual's life. The level of severity is categorized using score ranges, mild or no impact (≤49), some impact (50–55), substantial impact (56–59), and severe impact (60–78). Only individuals with a HIT score of 56 or more were recruited into the study. Several studies have defined a meaningful (clinically significant) change in the HIT score. Depending on the study design, subject pool and the type of analysis, a clinically significant change was defined as a change in the symptom score of at least 2.5 points.^27^^,^^28^

#### Study Lenses

Two lenses were used in the current study. (1) The Shamir Autograph II single vision or PAL with crizal rock was used as both subject's updated refractive prescription lens after visit 1 and the control lens that was either dispensed at visit 2 or 3 depending on the randomization. The control lens had no prism correction. Both the subject and the primary investigator were not aware that the updated prescription lens and the control lens were the same. (2) NL was the treatment lens either dispensed at visit 2 or 3 after randomization. The coating provided was similar to the one on the control lens. This ensured that the two lenses looked similar during the study and did not induce any bias in the subject's response.

#### Study Procedures

**Figure 1.**
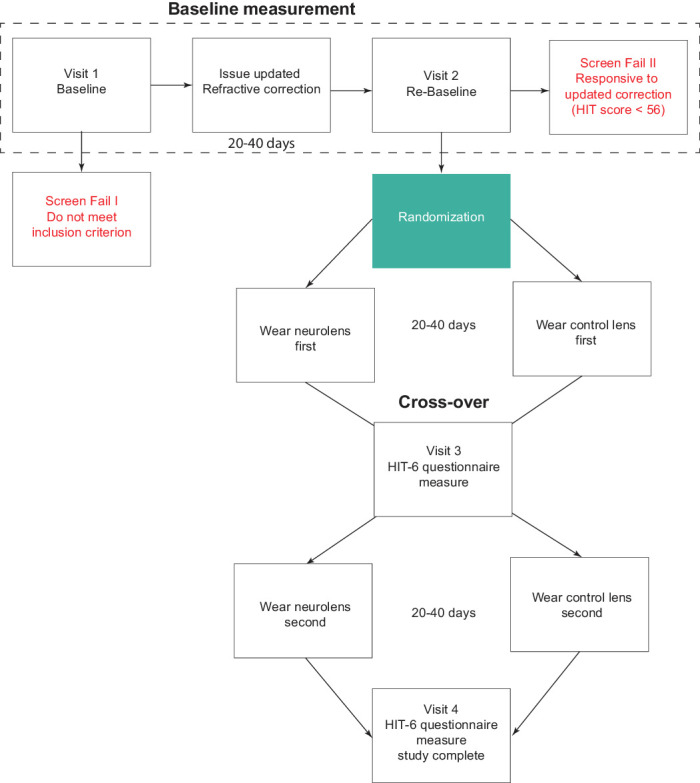
Flowchart indicating the subject journey from initial baseline visit to study completion.

**Figure 2.**
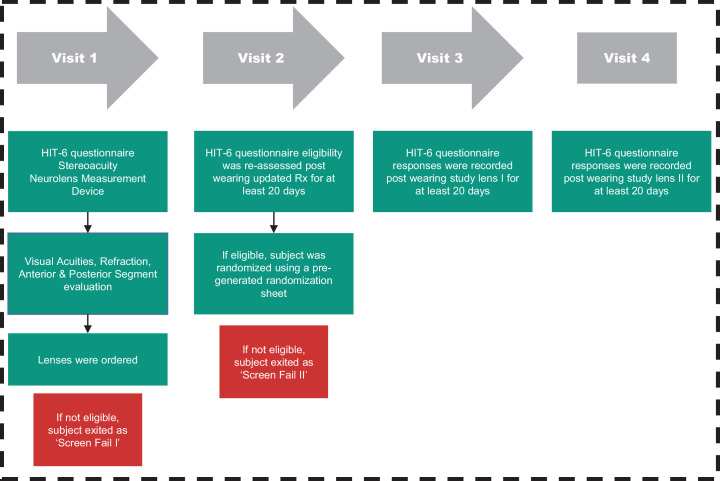
Flowchart indicating all the measurements and procedures performed at each visit.

Study subjects initially reviewed and signed an institutional review board–approved consent form. The investigator, or an authorized technician, then signed the consent form and filed it. The subject was then assigned an identification number and was only identified using this ID number thereafter. Study subjects completed four visits, that were separated by 30 ± 10 days. The study process and the procedures performed at each visit are summarized in Figures 1 and 2, and in the (Table A1).

At visit 1, baseline clinical (optometric) measurements were performed by masked clinicians. Measurements included visual acuities at both distance and near, stereopsis, NL measurement, fusional reserves, near point of accommodation and NPC, slit lamp examination, dry eye test (tear film break-up time), intraocular pressure, and fundus evaluation. All the study procedures are described in (Table A1). If a subject did not meet all of the inclusion criteria including a HIT score of at least 56 or met any exclusion criteria or (Table 1), they were excluded from the study and considered a visit 1 screen fail. If the subject met all the inclusion criteria and did not meet any exclusion criteria, the subject was assigned to subgroup 1 (prepresbyopic/young adults) or subgroup 2 (presbyopes/older adults who need additional near vision correction) and issued an updated refractive prescription. The subject was then scheduled for visit 2, within 30 ± 10 days after receiving their updated prescription glasses. Subjects’ participation in the study ended if during the study they had any change in their medications that the primary investigator deemed could impact the severity of a subjects’ headache symptoms.

Subjects were initially provided with an updated prescription to see if the symptoms that they experienced were relieved by providing an updated refractive correction. This period was defined as the run-in phase. Subjects were advised to wear spectacles for at least 8 hours a day. At visit 2, subjects’ symptoms were reassessed using the HIT questionnaire. If the subject had a score of less than 56 or had a significant change that met any exclusion criteria, their study participation ended and was considered a visit 2 screen fail. If the subject met all the inclusion criteria and did not meet any exclusion criteria, the subject was randomized by the optician, the only unmasked investigator at each site, using a pregenerated randomization sheet for the initial issue of control or treatment lenses. The pregenerated randomization sheet contained a subject ID column and the randomization order for the test lenses for each subject. A permuted block design approach was used for the randomization process using Microsoft Excel. This step was done separately for each site. The principal investigator and any research staff taking direct measurements remained masked as to which lenses the subject was issued at this visit. Also, all the subjects remained masked as to which lenses they received on this visit. Randomized subjects were then scheduled for the next visit, within 30 ± 10 days after visit 2. Given that visit 2 (run-in phase) was meant to minimize the impact of uncorrected refractive errors on the HIT score, data from this visit were considered as the subject's baseline symptom score.

At visit 3, the subject's symptoms were assessed again using the HIT-6 questionnaire. An unmasked optician swapped the lenses from control to treatment or vice versa and issued them to the subject. The principal investigator, the subject, and any research staff taking direct measurements remained masked as to which lenses the subject wore at this visit. Subjects were advised to wear spectacles with study lenses for at least 8 hours a day.

At the final visit 4, the subject's symptoms were reassessed using the HIT-6 questionnaire. All the measurement and questionnaire data were entered into an electronic database throughout the four visits.

#### Outcome Measurements

The primary end point was the difference between the subject's self-completed HIT-6 scores between NLs and control lenses. The secondary end point was the difference between the subject's self-completed HIT-6 scores between NLs and control lenses in a subgroup of subjects with reduced NPC (>5 cm).

### Sample Size Calculations

Sample size calculations were made based on an unpublished investigational study sponsored by Neurolens Inc. Data from a total of 87 individuals were analyzed. All the individuals had good stereopsis (≤50 arc sec). It was a cross-over study (similar to the current study) and the subjects included in the study wore both control and treatment lenses for 25 to 35 days. The effect size obtained along with the standard error was 0.19 ± 0.07 score points difference in the HIT questionnaire symptom improvement score between the two treatments (*z* score = 0.27; *P* = .01). For the current study, we have used 90% power and a 5% two-sided significance level to calculate the sample size required to achieve statistical significance. Based on the calculations using the software tool SAS, the minimum sample size required was 146. This study had a dropout rate of about 45% across different visits, primarily owing to improved symptoms after the run-in phase or subjects being lost to follow-up. Therefore, to obtain the required sample size with a dropout rate of approximately 50%, we recruited 300 subjects for this current study.

### Data Analysis

As summarized in the protocol, comparisons between the two treatments (NL and control) were evaluated based on the appropriate paired analyses. A modified intent-to-treat (ITT) analysis approach was used to analyze primary and secondary end points.^29^ Missing data were ignored, except for imputation analysis for the primary end point as detailed elsewhere in this article. Imputation served as our sensitivity analysis. Although it was outlined in the protocol submitted before the launch of the study, the study did violate two principles of a typical ITT analysis. First, we ended subject participation if they had a change in their headache medication during the study duration. The primary investigators at each site, who are licensed optometrists, used their professional discretion about the inclusion or exclusion of the patient when a medication change happened during the trial. The sponsors had no influence and optometrists, based on their clinical expertise, ended the subject's participation if they deemed that the medication change could impact the overall treatment outcome on headaches. Missing data from these subjects were assumed to be missing at random and an imputation analysis was used to assess the impact. Second, some participants had their randomized treatment assignment switched because they significantly scratched or chipped their updated prescription lenses that were supposed to serve as control lenses. This factor violated the ITT principle and led to unequal randomization. This occurrence was uncommon and happened in less than 5% of the study sample. The key issue was that the unanticipated damage of some patients’ updated refraction lenses (the control) made the lens recognizable to the patient. This factor would ultimately impact the masked nature of the study, given that the updated refractive prescription provided after visit 1 was reused as the control lens at either visit 3 or 4. If randomization was preserved in these cases, subjects would have been provided with a recognizable control lens or deviate from the protocol by increasing the wear period of the control while the lens was remade, which would not have been possible while keeping the practitioner masked. Therefore, in the interest in preserving the masked nature of the study, the randomization was switched in these cases, and the unmasked optician placed an order to remake the control lenses. Neither the subject nor the masked investigators were aware of this switch. The impact of the order and sequence of the treatment was performed to quantify the impact and/or significance of the treatment swap. Statistical significance was considered when the *P* value was measured at 0.05 or less. Two independent biostatisticians were employed to analyze the symptom score data and design the data analysis plan. They had no conflicts of interest. SAS (SAS Institute, Cary, NC) was used for statistical analysis. To test the normality of the baseline HIT scores, Shapiro–Wilk test was used. The Shapiro–Wilk test of the normality of the baseline scores (*n* = 170) results indicated significant evidence of non-normality (W = 0.969; *P* = 0.0007). Concluding that non-normality was present, a nonlinear mixed model (NLMM) analysis was warranted.

The primary end point was initially analyzed using the following linear mixed model (LMM) for repeated measures.

(1) $$y_{ijkl}=\mu +\alpha h_{i}+\beta_{j}+\gamma_{k}+\tau_{l}+u_{i\left(j\right)}+{ɛ}_{ijkl},$$

where

*y_ijkl_* is the HIT-6 score of the *i*^th^ subject randomized to the *j*^th^ treatment sequence at visit *k* experiencing treatment *l*;
µ is the overall model intercept;
*h* is the baseline HIT-6 score covariate with coefficient α;
β is a fixed effect for sequence;
γ is a fixed effect for period;
τ is a fixed effect for treatment;
*u_i_* is a random effect for subject *i*; and
ε*_ijkl_* is the residual.

We assumed *u_i_* ∼ *N* (0, *σ_u_^2^*) with compound symmetry covariance structure, and ε*_ijkl_* ∼ *N* (0, *σ_ε_^2^*). We fit a LMM (PROC MIXED in SAS) with HIT scores at each treatment visit as the dependent variable with fixed effects for baseline HIT, treatment, the sequence to which the participant was assigned (NL then control or control then NL) and period (visit 2 or visit 3), as well as a random intercept (or repeated effect) for study participant to account for the repeated measurements as covariates. The robustness of the model fit on the data was evaluated by assessing the normality of the residuals and by performing influence diagnostics which evaluated the influence of outliers (if any) on the overall model.

**Figure A1.**
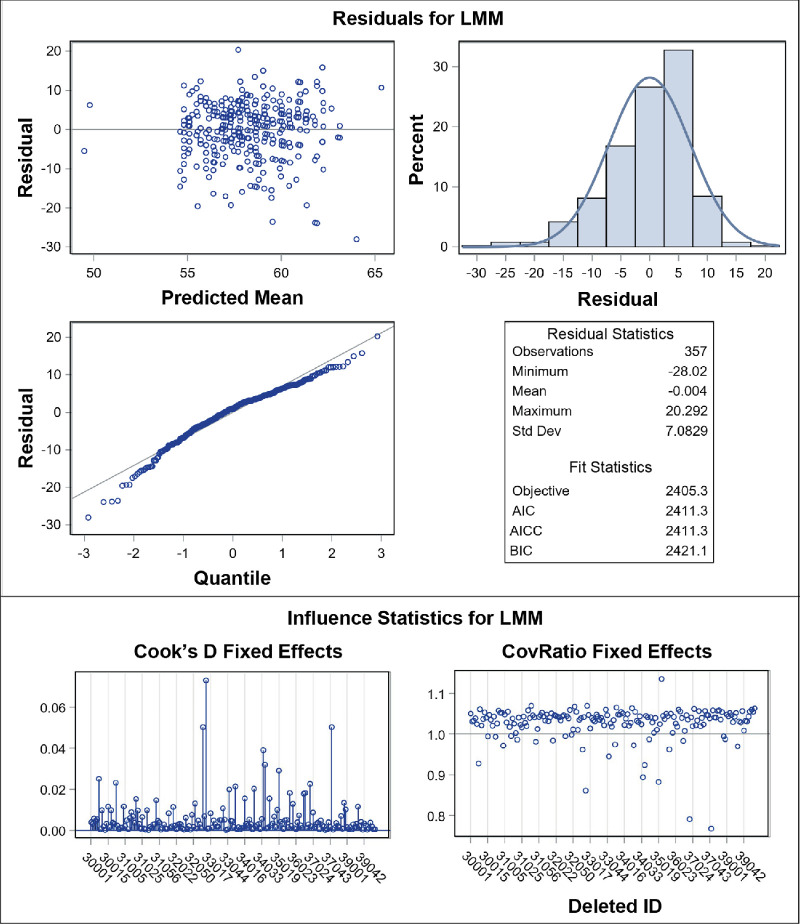
Residual plots and influence diagnostics for the LMM fit.

As shown in Figure A1, the analysis indicated a negative skew for the residuals with potential outliers influencing the data in one direction. Cook's Distance and Covariance ratio analysis indicated that there were a few individual data points (outliers) which exhibit high leverage over the model fit. Therefore, based on the results from the Shapiro–Wilk test and influence diagnostics, the data were fit with a robust regression model to minimize the impact of the outliers on the overall outcome. We used a robust linear mixed model (RLMM) with the same structure as model (1), but assuming that the residuals follow a *t* distribution with *ν* degrees of freedom.^30^ For this procedure, we used PROC NLMIXED in SAS. The parameter estimates from the LMM were used to provide the starting values for the RLMM parameters. The starting value for the *t*-distribution degree of freedom was 30, to evaluate the normality assumption. The model converged successfully, and analysis outcomes are presented in the results section. In the ITT dataset, there were 11 subjects with missing values for visit 3, and 22 subjects with missing values for visit 4. As a sensitivity analysis, we used multiple imputation to evaluate the effect of these missing values on the results of RLMM analysis. We assumed that all the missing values were missing at random. Fifty imputation datasets were created using the multiple imputation procedure in SAS. Missing values at visit 3 and visit 4 were imputed using the baseline HIT-6 score for that subject, as well as other HIT-6 scores from other subjects for that visit, sequence, period, and treatment.

## Results

### Summary of the Baseline Data

Of the 300 subjects recruited, 195 subjects were randomized. A summary of the demographic and measurement data has been tabulated in Table 2. Of the 195 subjects, only 170 subjects (88%) completed all four visits. A total of 79 subjects were not symptomatic after wearing an updated refractive error correction for 30 ± 10 days, so their study participation ended as a visit 2 screen fail. Nineteen subjects were lost to follow-up, 8 of them after visit 1, 6 of them after visit 2, and 5 of them after visit 3. Two subjects had anticipated adverse events (one with control lens and one with treatment lens) wherein the subject was not able to adapt to or wear the study lenses. Eight subjects’ study participation was ended due to noncompliance, which included either insufficient lens wear time or inability to come back for the follow-up visit within the scheduling window. A breakdown of the other exits has been reported in Table A3. The final sample consisted of 122 young adults (single vision lenses) and 73 presbyopes (progressive addition lenses). Of the 195 subjects who were randomized, 112 subjects wore NL first, and 83 subjects wore control lens first at visit 2 and were then switched to the other pair of test lens at visit 3. There were a few reasons that caused this imbalance in the randomization numbers. Twelve subjects had their randomization swapped from control first to NL first owing to subject-related errors wherein their control lenses (updated refractive correction) were scratched significantly during the run-in period. These visible scratches or irregularities on the lens could have compromised the masked nature of the study. Therefore, lens order was switched to NL first and the control lens was reordered. Neither the masked investigator nor the subject was aware of the switch. Five subjects had their randomization swapped from NL first to control first owing to lab errors wherein the NLs were edged improperly and could not fit in the subjects’ frame. Finally, each site's randomizations numbers were split equally for 30 subjects. However, as reported in Table A2, not all sites recruited an equal number of subjects, with sites ranging from a maximum enrollment of 44 subjects to a minimum of 7 subjects. A breakdown of site enrollment and exit numbers are listed in Table A2. This imbalance in recruitment between the sites led to unequal randomization numbers.

**Table 2.** Summary of Demographic and Baseline Clinical Data of Participants Who Were Randomized

Parameter (*N* = 195)	Mean ± SD
Age, years	37.34 ± 10.85
Hours spent on near/digital work in a week, hours	47.36 ± 19.81
Spherical equivalent	−1.04 ± 1.75 D
Distance phoria – negative sign indicates exo deviation	−1.87 ± 1.16 PD
Near phoria – negative sign indicates exo deviation	−4.63 ± 2.33 PD
Near point of accommodation (in cm) – young adults only (*N* = 122)	11.0 ± 7.0
NPC (in cm)	8.0 ± 9.0
Base-in fusional reserves (break)	9.0 ± 7.0 PD
Base-out fusional reserves (break)	15.0 ± 8.0 PD
Prescribed prism power at distance (Neurolens value)	1.29 ± 0.53 PD

### Primary and Secondary Outcomes

**Figure 3.**
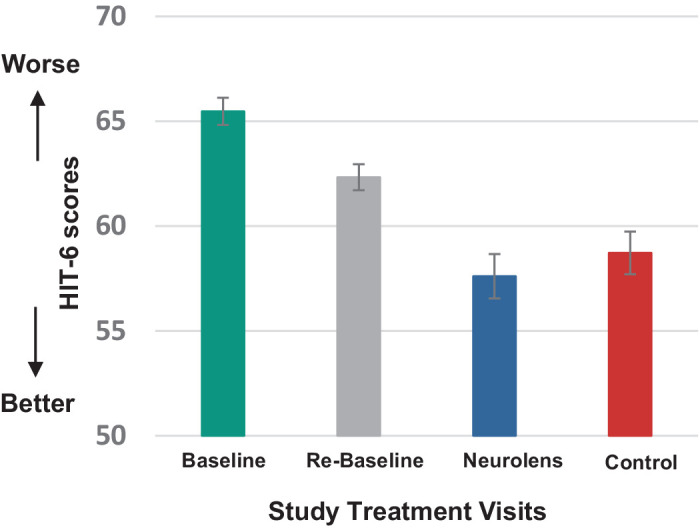
HIT-6 scores were plotted as a function the study visits and treatment lenses: NL, control (updated refractive correction), baseline score at visit 1 and re-baseline score at visit 2. The mean HIT score at baseline (visit 1) was 65 points. The mean HIT-6 score at re-baseline (visit 2) was 63 points and decreased to a mean of 60 points after control lens wear for 20 to 40 days and decreased to 58 points after wearing NL for 20 to 40 days. The error bars indicate the 95% CIs.

We fit a RLMM (PROC NLMIXED in SAS) with HIT scores at visit 2 and visit 3 as the dependent variable with fixed effects for baseline HIT, treatment, the sequence to which the participant was assigned (NL then control or control then NL) and period (visit 2 or visit 3), as well as a random intercept (or repeated effect) for study participant to account for the repeated measurements as covariates. The analysis outcomes are tabulated in Table 3. Figure 3 represents the means (and 95% confidence intervals [CIs]) of the HIT score of the overall sample at each visit/ treatment (mean difference between the treatments, −1.53 points; 95% CI, −2.8 to −0.26; *P* = 0.*01*). Overall, subjects reported a statistically significant decrease in their symptom score when they wore the NL.

**Table 3.** Summary of Results From the RLMM Fit on the ITT Data (*N* = 195)

Parameter	Estimate (NL-Control)	*t* Value	*P* Value	95% Confidence Limits	
Baseline HIT-6 score	0.5283	5.92	*<0.0001* ^a^	0.3522	0.7044
Treatment	−1.5357	−2.39	*0.0179* ^a^	−2.8042	−0.2671
Period	−1.0903	−1.69	*0.0928*	−2.3635	0.1829
Sequence	−0.8051	−1.03	*0.3047*	−2.3483	0.7381

a Indicates statistical significance (*P* < 0.05).

We used multiple imputation to evaluate the effect of these missing values on the results of RLMM analysis. The results are tabulated in Table 4 below. Similar to Table 3, RLMM was fitted for the 50 imputed datasets with HIT scores at visits 3 and 4 as the dependent variable with fixed effects for baseline HIT, treatment, the sequence to which the participant was assigned (NL then control or control then NL) and period. As described in the protocol, the RLMM was also fit in the subgroup of subjects with an NPC of more than 5 cm (*n* = 98 subjects). The results are shown in Table 5 (mean difference, −1.89 points; 95% CI, −4.27 to −0.47; *P* = 0.*11*). Overall, subjects reported a greater decrease in their symptom score when they wore NL compared with the control lens, but it was not statistically significant.

**Table 4.** Summary of the Sensitivity Analysis Results From the RLMM Fit After Multiple Imputation

Parameter	Estimate (NL-Control)	*t* Value	*P* Value	95% Confidence Limits	
Baseline HIT-6	0.5175	5.79	<0.0001^a^	0.3422	0.6928
Treatment	−1.4804	−2.14	*0.03* *27* ^a^	−2.8386	−0.1222
Period	−1.1019	−1.59	0.1118	−2.4604	0.2567
Sequence	−0.8622	−1.11	0.2681	−2.3885	0.6640

a Indicates statistical significance (*P* < 0.05).

**Table 5.** Summary of the Results From the RLM Model Fit on Subjects With Reduced NPC (*N* = 98)

Parameter	Estimate (NL-Control)	*t* Value	*P* Value	95% Confidence Limits	
Baseline HIT-6	0.5647	4.55	*<0.0001* ^a^	0.3180	0.8114
Treatment	−1.8995	−1.59	*0.1163*	−4.2787	0.4797
Period	−0.4995	−0.42	*0.6773*	−2.8756	1.8765
Sequence	−1.9994	−1.64	*0.1054*	−4.4272	0.4284

a Indicates statistical significance (*P* < 0.05).

## Discussion

The current randomized, placebo-controlled, cross-over study demonstrated that NL produced a statistically significant improvement in the headache symptom scores compared with a control lens. Although the study did find a symptom improvement with NL in the subgroup with a decreased NPC, this improvement did not achieve statistical significance. The mean difference in the HIT score change between the two treatment groups was 1.53 points. Based on the CIs of the mean difference in HIT score improvement, the difference in the symptom relief between the two treatment groups could be as large as 2.8 points. Therefore, a clinically meaningful improvement with NL treatment compared with placebo cannot be ruled out with high certainty in the current study.

Optical tints have been proposed previously to decrease headache frequency and improve quality of life in patients with migraine-related headaches.^31^ Blue light filter coatings, often prescribed by clinicians worldwide for digital eye strain, have been reported to be ineffective in relieving CVS-related symptoms when compared with nonblue light filtering lenses.^32^^–^^36^ Previous placebo-controlled trials that evaluated the impact of base-in prisms on vision-related symptoms have reported contradictory results.^11^^,^^12^^,^^37^ Unlike the current study, which included anyone with symptoms, these studies purely focused on individuals with specific disorder known as convergence insufficiency diagnosed based on a specific criterion. They also used a different questionnaire, the convergence insufficiency symptom score, to assess the impact of the treatment. There were several reasons that could have led to the contradictory results, including the age group (children, young adults, or presbyopes), sample size, study design (parallel arm vs cross-over), methods used to prescribe prism (Sheards criterion vs fixation disparity), and wear time (2 weeks to 3 months). A recently published paper evaluated the efficacy and safety of interventions for treating eye strain related to computer use.^38^ The authors concluded that none of the commercially available spectacle lenses relative to a control lens were able to effectively relieve digital eyestrain related symptoms. In the current study, a cross-over design was used on a large sample of adults who were enrolled based on their self-reported headache symptoms. A prescribing guideline that took both the subject's dissociated phoria and fixation disparity into account was used. The current study found that NL outperformed the control lens in reducing the impact of digital eyestrain-related headache symptoms.

There is no consensus on the best prescribing guidelines for prisms, with some arguing that an associated phoria is more successful, whereas others use guidelines based on dissociated phoria measurements, such as the Sheard's criterion.^39^^,^^40^ Previous studies did report that the magnitude of the clinical measurements including Sheard's criterion or heterophoria do not correlate with the symptoms experienced.^23^ Thus, these studies by using a specific criterion may have overlooked individuals with CVS that may not be classified as someone with convergence insufficiency or otherwise. Therefore, the current study used a rather simple inclusion and exclusion criteria based primarily on symptoms and not on their clinical measurements, such as the magnitude of phoria or any specific criterion such as Sheard's criterion. With the NL process, individuals were identified using a simple questionnaire and were then measured using an objective technique that takes into account both dissociated phoria and associated phoria measurements. The proprietary algorithm then uses an iterative algorithm to provide a prescribing guideline called the Neurolens value. Previous studies also prescribed prisms only as reading glasses and not something that could be worn full time. This factor could have resulted in compliance issues that may have been under-reported. With the NL contoured design, subjects wore one pair of lenses that worked for both distance and near without the need to switch glasses based on the distance the subject was viewing.

The current study is a first of a kind double-masked cross-over study involving spectacle lenses to help patients with vision-related headaches. Unlike typical placebo-controlled studies, masking patients and investigators, tracking wear time and compliance, and assessing the true impact of the treatment without an influence of any external variables such as temporary or over-the-counter pharmacological interventions given the long study duration is particularly challenging for a spectacle lens intervention and was a focus of the study administration. The current study, although outlined in the protocol, did violate two ITT principles. The two ITT violations of the study include ending study participation if or when the subject had a change in their headache medication and swapping subject's treatment randomization order when the updated refractive lens was significantly scratched or damaged. Obviously, taking new or changing an existing medication that is proven to affect headaches can skew the study outcomes. The primary investigators at each site, who are licensed ODs, used their professional discretion about the inclusion or exclusion of the patient when a medication change happened during the trial. As reported in Table A3^8^,^9^, there were four such subjects; one was a screen fail at visit 2. Of the three subjects who were randomized, two completed all four visits and their inclusion in the ITT analysis did not affect the overall outcome of the study. The other subject, however, started taking pain medications while wearing the control and was exited at visit 3 before wearing the treatment lens. The subject would likely have biased the results because they were using an additional treatment for headaches. When included in the ITT analysis, this difference did not affect the overall conclusion. The second ITT violation was the change in the order of the treatment cross-overs owing to heavy scratches or lens damage, which occurred in less than 5% of the study sample. In retrospect, an extra set of spectacle lenses for each group to act as a replacement in anticipation of damage to the original lens pair would have addressed the situation. This factor, therefore, was a design limitation. However, providing damaged lenses would have compromised the masked nature of the study. Any efforts to preserve randomization in these cases we would have needed to either give the subject a recognizable control lens or deviate protocol by increasing the wear period of the control while the control lens was remade, which would not have been possible while keeping the investigators masked. Another option would have been to exit the patient from the study, which would also be an ITT violation. For these reasons, the unmasked study administrator and the unmasked optician at the site chose to switch the treatment arm of 12 patients. The masked investigators or the subjects were not aware of the switch; therefore, no bias was introduced. The impact of the order or sequence of the treatment was performed to assess the impact of this change and as reported found it to be limited. Therefore, the two violations had minimal to no impact on the study integrity and outcomes. The current study also had no washout period between the two treatments between visits 3 and 4. However, given that there was no sequence or order effect on the overall outcome, we believe that the impact of carry-over was minimal. The wear time for each treatment was also in line with a previous study that showed that 3 weeks or more of a wear time is sufficient to see the impact of a particular optical treatment.^37^ Subject compliance was also not monitored in the study. However, given that most of our subjects needed refractive correction, it is likely that they wore the study lenses most of the time for the sake of visual clarity.

## Conclusions

Headaches and eye strain are significant concerns in the current digital world. The Neurolens process, an objective measurement technique coupled with the contoured lens design demonstrated a statistically significant improvement in the quality of life of individuals impacted by headaches versus a placebo treatment in this double-masked cross-over study. Although the overall magnitude of the reduction was not clinically significant, a clinically meaningful improvement with Neurolens treatment cannot be ruled out with high certainty in the current study. The study also warrants evaluation and treatment of vision-related disorders in patients significantly impacted by headaches or digital eyestrain who may not fit the existing diagnostic criterion for binocular vision issues.

## Appendix

**Table A1.** Summary of Clinical and Research Measurements and Procedures During Each Visit

Parameter	Measurement Protocol
**Visit 1**	
Visual acuity	Distance visual acuity was measured using a Snellen or equivalent visual acuity chart at a testing distance of 20 feet, either in a mirrored room or a long, 20-foot lane. Near visual acuity is tested using a Snellen or equivalent near visual acuity chart at 40 cm.
Objective and subjective refraction	Objective refraction was done using an autorefractor. Subjective refraction was performed using the maximum plus/ minimum minus best visual acuity criteria.
NL measurement for heterophoria (based on the manufacturer's user manual)	The NL Measurement Device uses an eye tracking system along with a stereoscopic display to measure eye alignment at distance (6 m) and at near (50 cm). It is a 3-minute measurement with four major measurement aspects involved. There are two types of targets presented during the measurement, a central fixation target (yellow fixation cross) and an actively moving peripheral target (planets and stars). The subject viewed the central yellow fixation cross throughout the 3-minute measurement.
	PD measurement
	The subjects’ PD is initially measured. The displays are automatically adjusted to match the subjects’ pupillary distance, so the center of the display (fixation cross) coincides with the pupil center for each eye.
	Base alignment test (dissociated phoria)
	Fusable targets, both central and peripheral targets, are presented at 6m based on the subjects’ measured PD. Similar to a dissociated phoria test, the eyes are then dissociated using nonfusable targets (fixation cross in one eye and a rule with 6 Petri dishes filled with dots in the other eye). The tracking algorithm then waits for the eyes to stabilize, and the direction of gaze will be then measured.
	Eye conditioning
	A diverging peripheral target routine was then presented to both eyes. These images are difficult to fuse and according to the manufacturer, it is done to relax accommodation and vergence mechanisms.
	Fine alignment (associated phoria)
	Peripheral fusional targets are then placed at the gaze position measured during the base alignment. Similar to a fixation disparity test, the central fixation cross is presented to one eye at a time and the geometric position of the yellow cross is adjusted until the eye movement (if any) was neutralized. This is printed out as the misalignment value at that viewing distance.
	This procedure is repeated at distance (6 m) and near (50 cm) viewing distances. The device then takes phoria measurements at both distance and near and calculates the NL value, a prescribing guideline which represents the prism correction at distance. The device also calculates the subject's gradient AC/A ratio.
Headache-related symptoms	Headache related symptoms were assessed using the validated HIT-6 questionnaire
Stereoacuity	Randot circles in the randot stereoacuity and the stereo testing glasses were used according to the manufacturer's instructions. The appropriate near correction was used.

**Table A1.** Continued

Parameter	Measurement Protocol
Near point of accommodation	The near point of accommodation was measured using a 20/30 letter target on the Royal Airforce Rule (RAF). Measurements were repeated three times and the mean value was recorded.
Near point of vergence (NPC)	The NPC was measured using a 20/30 letter target on the Royal Airforce Rule (RAF). Measurements were repeated three times and the mean value was recorded.
Fusional vergence	Fusional vergence was measured using base-out prisms (positive fusional vergence) and base-in prisms (negative fusional vergence) of a prism bar using the step method. The target used was a 20/30 letter on the distance chart with the updated prescription in place.
Lens orders and dispensing	Two pairs of lenses were ordered. One was a refractive correction with no prism served as control lens and the updated refractive correction (Shamir Optical's Autograph II lens). The second pair was a NL which had refractive correction plus the prism correction (based on the NL value). Subject was initially dispensed updated refractive correction (Shamir Optical's Autograph II lens) and had to wear it for ≥20 days before they come in for visit 2
**Visit 2**	
Headache-related symptoms	Headache related symptoms were assessed using the validated HIT-6 questionnaire
Randomization	The subject was randomized by the optician using a pregenerated randomization sheet. The optician was the only unmasked investigator at each site. The permuted block design approach was applied for the randomization process using Microsoft Excel. This was done separately for each site.
**Visit 3**	
Headache-related symptoms	Headache-related symptoms were assessed using the validated HIT-6 questionnaire. Treatment lenses were switched based on the randomization protocol.
**Visit 4**	
Headache-related symptoms	Headache-related symptoms were assessed using the validated HIT-6 questionnaire

**Table A2.** A Breakdown of Site Enrollment, Randomization, Exit, and Study Completion Numbers From Each Site

Site Number	Enrolled	Screen Failures	Randomized	Dropouts	Completed
Site 30	23	2	21	2	19
Site 31	44	17	27	3	24
Site 32	34	7	27	6	21
Site 33	31	10	21	1	20
Site 34	32	6	26	0	26
Site 35	23	12	11	3	8
Site 36	16	6	10	3	7
Site 37	47	18	29	3	26
Site 38	7	4	3	1	2
Site 39	43	23	20	3	17
Total	300	105	195	25	170

**Table A3.** Patients Whose Participation Was Ended During the Study Period Along With the Reason for the Exit

Subject ID	Last Visit Completed	Reason for Exit	Randomized (Y/N)
31009	2	V2 HIT6 <56	N
31011	2	V2 HIT6 <56	N
31014	2	V2 HIT6 <56	N
31028	2	V2 HIT6 <56	N
31032	2	V2 HIT6 <56	N
31035	2	V2 HIT6 <56	N
31036	2	V2 HIT6 <56	N
31037	2	V2 HIT6 <56	N
31038	2	V2 HIT6 <56	N
31041	2	V2 HIT6 <56	N
31042	2	V2 HIT6 <56	N
31044	2	V2 HIT6 <56	N
31046	2	V2 HIT6 <56	N
31049	2	V2 HIT6 <56	N
31050	2	V2 HIT6 <56	N
31053	2	V2 HIT6 <56	N
32006	2	V2 HIT6 <56	N
32013	2	V2 HIT6 <56	N
32026	2	V2 HIT6 <56	N
32028	2	V2 HIT6 <56	N
32035	2	V2 HIT6 <56	N
32038	2	V2 HIT6 <56	N
33004	2	V2 HIT6 <56	N
33006	2	V2 HIT6 <56	N
33010	2	V2 HIT6 <56	N
33011	2	V2 HIT6 <56	N
33016	2	V2 HIT6 <56	N
33019	2	V2 HIT6 <56	N
33032	2	V2 HIT6 <56	N
33034	2	V2 HIT6 <56	N
33042	2	V2 HIT6 <56	N
33049	2	V2 HIT6 <56	N
34004	2	V2 HIT6 <56	N
34013	2	V2 HIT6 <56	N
34024	2	V2 HIT6 <56	N
34032	2	V2 HIT6 <56	N
34038	2	V2 HIT6 <56	N
35001	2	V2 HIT6 <56	N
35004	2	V2 HIT6 <56	N
35011	2	V2 HIT6 <56	N
35015	2	V2 HIT6 <56	N
35018	2	V2 HIT6 <56	N
35027	2	V2 HIT6 <56	N
35038	2	V2 HIT6 <56	N
36026	2	V2 HIT6 <56	N
37003	2	V2 HIT6 <56	N
37005	2	V2 HIT6 <56	N
37010	2	V2 HIT6 <56	N
37011	2	V2 HIT6 <56	N
37014	2	V2 HIT6 <56	N
37017	2	V2 HIT6 <56	N
37020	2	V2 HIT6 <56	N
37022	2	V2 HIT6 <56	N
37025	2	V2 HIT6 <56	N
37027	2	V2 HIT6 <56	N
37038	2	V2 HIT6 <56	N
37041	2	V2 HIT6 <56	N
37045	2	V2 HIT6 <56	N
37048	2	V2 HIT6 <56	N
37058	2	V2 HIT6 <56	N
38002	2	V2 HIT6 <56	N
38015	2	V2 HIT6 <56	N
38030	2	V2 HIT6 <56	N
39003	2	V2 HIT6 <56	N
39005	2	V2 HIT6 <56	N
39006	2	V2 HIT6 <56	N
39012	2	V2 HIT6 <56	N
39014	2	V2 HIT6 <56	N
39016	2	V2 HIT6 <56	N
39021	2	V2 HIT6 <56	N
39026	2	V2 HIT6 <56	N
39028	2	V2 HIT6 <56	N
39033	2	V2 HIT6 <56	N
39040	2	V2 HIT6 <56	N
39044	2	V2 HIT6 <56	N
39046	2	V2 HIT6 <56	N
39048	2	V2 HIT6 <56	N
39049	2	V2 HIT6 <56	N
39061	2	V2 HIT6 <56	N
30008	1	Lost to follow-up	N
30017	2	Lost to follow-up	Y
31023	3	Lost to follow-up	Y
31027	3	Lost to follow-up	Y
31029	1	Lost to follow-up	N
31047	2	Lost to follow-up	Y
32004	3	Lost to follow-up	Y
32019	1	Lost to follow-up	N
32024	2	Lost to follow-up	Y
32033	2	Lost to follow-up	Y
32050	3	Lost to follow-up	Y
36014	2	Lost to follow-up	Y
36079	2	Lost to follow-up	Y
37018	1	Lost to follow-up	N
37036	3	Lost to follow-up	Y
38026	1	Lost to follow-up	N
39017	1	Lost to follow-up	N
39055	1	Lost to follow-up	N
39056	1	Lost to follow-up	N
34039	1	Noncompliance	N
35002	2	Noncompliance	Y
35005	1	Noncompliance	N
35028	1	Noncompliance	N
35030	1	Noncompliance	N
36012	1	Noncompliance	N
36013	1	Noncompliance	N
38018	3	Noncompliance	Y
30026	1	Study lens not cut correctly	N
35032	1	Study lens not cut correctly	N
35034	1	Study lens not cut correctly	N
36023	3	Study lens not cut correctly	Y
39042	3	Study lens not cut correctly	Y
39047	3	Study lens not cut correctly	Y
32029	2	Change in eyeglass RX	Y
37050	2	Change in eyeglass RX	Y
39037	2	Change in eyeglass RX	N
39053	1	Change in eyeglass RX	N
39063	1	Change in eyeglass RX	N
39067	1	Change in eyeglass RX	N
30023	3	Change in medication	Y
32023	4	Change in medication	Y
33009	4	Change in medication	Y
37008	1	Change in medication	N
36002	1	Wrong pair dispensed after V1	N
36003	1	Wrong pair dispensed after V1	N
36011	1	Wrong pair dispensed after V1	N
37042	2	Subject discontinued participation	Y
37057	2	Subject discontinued participation	N
35006	3	Anticipated AE	Y
39011	2	Anticipated AE	Y
35022	4	Missing V2 survey data	Y

**Table A4.** HIT-6 Questionnaire, Which Was Administered at Every Visit

Questions	Never		Rarely		Sometimes		Very often		Always	
When you have headaches, how often is the pain severe?										
How often do headaches limit your ability to do usual daily activities including household work, work, school, or social activities?										
When you have a headache, how often do you wish you could lie down?										
In the past 4 weeks, how often have you felt too tired to do work or daily activities because of your headaches?										
In the past 4 weeks, how often have you felt fed up or irritated because of your headaches?										
In the past four weeks, how often did headaches limit your ability to concentrate on work or daily activities?										
How completely do the questions you just completed characterize your symptoms?	Multiple choice (1–10) – 1 not at all, 10 completely									
	1	2	3	4	5	6	7	8	9	10
